# DWI-based Biologically Interpretable Radiomic Nomogram for Predicting 1-year Biochemical Recurrence after Radical Prostatectomy: A Deep Learning, Multicenter Study

**DOI:** 10.2174/0115734056403104250527045320

**Published:** 2025-06-10

**Authors:** Xiangke Niu, Yongjie Li, Lei Wang, Guohui Xu

**Affiliations:** 1 Department of Interventional Radiology, Sichuan Cancer Hospital and Institute, Sichuan Cancer Center, School of Medicine, University of Electronic Science and Technology of China, Chengdu 610041, China; 2 Department of Radiology, Affliated Hospital of Chengdu University, Chengdu 610081, Sichuan, China; 3 MOE Key Lab for Neuroinformation, University of Electronic Science and Technology of China, Chengdu 610054, China; 4 Department of Radiology, Ninety-three Hospital, Jiangyou City 621700, Sichuan, China

**Keywords:** Deep learning, Diffusion-weighted imaging, Radiomic, Biochemical recurrence, Prostate cancer, Tumor microenvironment

## Abstract

**Introduction:**

It is not rare to experience a biochemical recurrence (BCR) following radical prostatectomy (RP) for prostate cancer (PCa). It has been reported that early detection and management of BCR following surgery could improve survival in PCa.

This study aimed to develop a nomogram integrating deep learning-based radiomic features and clinical parameters to predict 1-year BCR after RP and to examine the associations between radiomic scores and the tumor microenvironment (TME).

**Methods:**

In this retrospective multicenter study, two independent cohorts of patients (n = 349) who underwent RP after multiparametric magnetic resonance imaging (mpMRI) between January 2015 and January 2022 were included in the analysis. Single-cell RNA sequencing data from four prospectively enrolled participants were used to investigate the radiomic score-related TME. The 3D U-Net was trained and optimized for prostate cancer segmentation using diffusion-weighted imaging, and radiomic features of the target lesion were extracted. Predictive nomograms were developed *via* multivariate Cox proportional hazard regression analysis. The nomograms were assessed for discrimination, calibration, and clinical usefulness.

**Results:**

In the development cohort, the clinical-radiomic nomogram had an AUC of 0.892 (95% confidence interval: 0.783--0.939), which was considerably greater than those of the radiomic signature and clinical model. The Hosmer–Lemeshow test demonstrated that the clinical-radiomic model performed well in both the development (*P* = 0.461) and validation (*P* = 0.722) cohorts.

**Discussion:**

Decision curve analysis revealed that the clinical-radiomic nomogram displayed better clinical predictive usefulness than the clinical or radiomic signature alone in both cohorts. Radiomic scores were associated with a significant difference in the TME pattern.

**Conclusion:**

Our study demonstrated the feasibility of a DWI-based clinical-radiomic nomogram combined with deep learning for the prediction of 1-year BCR. The findings revealed that the radiomic score was associated with a distinctive tumor microenvironment.

## 1 INTRODUCTION

### 1.1 Background

Radical prostatectomy (RP) is a primary treatment for localized prostate cancer (PCa). The literature indicates that the 3- to 5-year biochemical recurrence (BCR) risk post-RP is approximately 28–34% [1]. Nearly two-thirds of these instances occurred during the first two years [2, 3]. Men with BCR are more likely to develop metastatic disease and die from PCa progression than men without BCR [4]. Early prediction of BCR following surgery can be useful for identifying patients who may benefit from adjuvant therapy, which in turn improves survival in PCa patients [5, 6]. Preoperative prostate-specific antigen (PSA) levels, Gleason grade, and tumor node metastasis classification (TNM) stage have all been identified as risk factors for BCR in PCa patients. The models for estimating the BCR include the Cancer of the Prostate Risk Assessment (CAPRA) score, Han tables, and Kattan nomogram, which are prominent in clinical practice [7, 8]. Notably, multiparametric magnetic resonance imaging (mpMRI) enhances the predictive power of these models [9].

### 1.2 Problem Statement

High-b value diffusion-weighted imaging (DWI) is considered a crucial sequence according to the most recent version of the Prostate Imaging-Reporting and Data System version 2.1 (PI-RADS 2.1). This is because DWI may improve the identification and localization of PCa, which is clinically significant [10]. Many studies have examined the connections between lesion aggressiveness and DWI-related metrics, such as extraprostatic extension and the Gleason score (GS) [11, 12]. Radiomics is a growing field of quantitative image analysis that seeks to connect extracted large-scale image information with clinical and biological objectives. However, the associations of individual prognostic radiomic signatures with specific biological pathways and how those pathways influence radiomic risk classification remain unclear.

### 1.3 Motivation and Objectives

Over the past decade, the identification and segmentation of PCa *via* MRI have become challenging and laborious tasks [13, 14]. In recent years, the segmentation of medical images has been revolutionized by deep learning (DL) [15, 16]. The U-Net algorithm is a convolutional neural network (CNN) that is widely used in deep learning. This algorithm has demonstrated potential for identifying, segmenting, and categorizing prostate cancer on MR images [17, 18]. Owing to their superior performance in medical image segmentation, researchers have developed a variety of updated algorithms over time. In terms of the 3D U-Net structure, deeper networks produced superior segmentation performance. Thus, this study aimed to develop a 3D U-Net-based radiomic model that uses preoperative DWI images to predict BCR after one year and to analyze the biological relationships of the radiomic score.

## 2 MATERIALS AND METHODS

### 2.1 Study patients

This retrospective multicenter study was conducted in accordance with the Declaration of Helsinki. A total of 424 consecutive men with clinically suspected PCa who underwent RP between January 2015 and January 2022 were included in the institutional review board-approved databases of the development cohort (Institution I, n=221) and the validation cohort (Institution II, n=203). The patients or their legal representatives provided informed consent to participate in this study. The inclusion criteria were as follows: (1) underwent RP following mpMRI, (2) had no history of neoadjuvant or adjuvant therapy, (3) had high-quality images with visible lesions, and (4) had follow-up information, including postoperative serum PSA levels. The exclusion criteria were as follows: (1) no DWI images, (2) low-quality MR images, (3) PSA persistence, (4) missing clinical data, and (5) insufficient follow-up to determine BCR. The patients underwent a digital rectal examination, and serum PSA tests after one month of RP, every three months for the first two years, and every six months beginning in the third year. If PSA values reach 0.2 ng/mL twice after surgery, BCR is confirmed [19]. Finally, 349 males were included in the analysis.

**Fig. (1):**
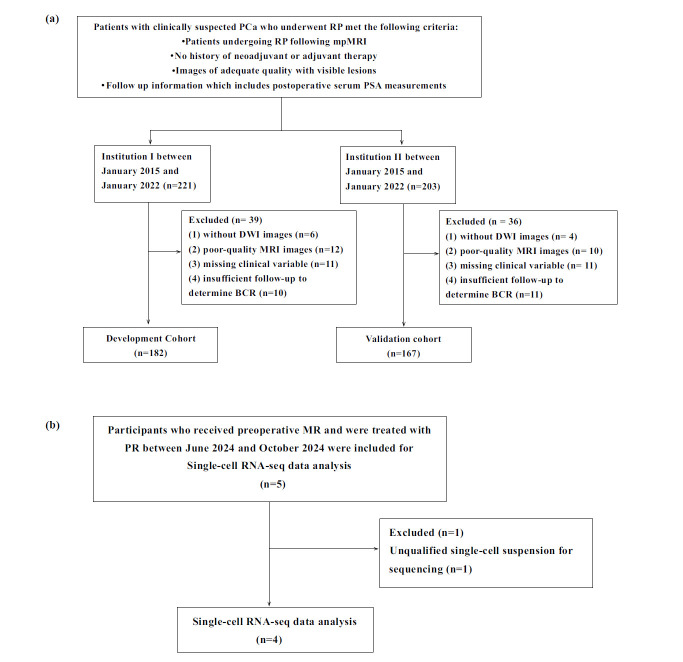
Study workflow and patient enrollment flowchart. (**a**) Development and validation cohorts; (**b**) Single-cell genomics sample.

To investigate the relationship between the radiomic score and the tumor microenvironment (TME), five participants who underwent RP following mpMRI in the development cohort from June 2024 to October 2024 were prospectively included. These individuals at the Institution I provided their informed consent. Fresh tumor tissues were collected and utilized to prepare single-cell suspensions. Sequencing was performed on a 10X Genomics platform. (Fig. **1a** and **b**) depicts the detailed patient recruitment procedure.

### 2.2 Manual Annotations

The methods used for DWI image acquisition are described in Table **S1**. Manual delineation was performed with 3D Slicer version 4.11 (Table **S2**). In the development dataset, one junior radiologist (with more than five years of experience in prostate MRI) manually identified all discernible target lesions on DWI images in the pelvic region (Mask 1). An expert radiologist (with over 15 years of experience in prostate MRI) subsequently modified the set of manual annotations (Mask 2). The Dice score was used to evaluate the reliability of manual annotations across masks.

### 2.3 Groundtruth Delineation

A 3D U-Net CNN (Table **S3**) was established for PCa segmentation in DWI images. For the development of the models, manual annotations of the target lesion on DWI were considered ground truth for segmentation assessment (Mask 2).

### 2.4 Segmentation Performance and Feature Extraction

The Dice similarity coefficient (DSC), volumetric overlap error (VOE), and relative volume difference (RVD) are metrics that are used to evaluate segmentation performance. Radiomic features were calculated *via* the open-source Python software PyRadiomics (version 2.2.0; https://pyradiomics.readthedocs .io/), as detailed in Table **S4**.

### 2.5 Development of the Radiomic Signature

The least absolute shrinkage and selection operator (LASSO) method [20] was used to select the most relevant and robust features from the development cohort. The radiomic score for each patient was obtained by multiplying the final retained characteristics with their coefficients. Patients in the development cohort were divided into groups with low- or high-risk scores on the basis of the median value of the radiomic score.

### 2.6 Comparison of Biological Differences between the Low- and High-risk Score Groups

Single-cell RNA sequencing (scRNA-seq) data collection and integration are described in Table **S5**. In Step 1, the Seurat package for R was used to transform the scRNA-seq data into Seurat objects. The FindAllMarkers functions were used to determine marker gene expression, followed by the SingleR package and the CellMarker dataset to identify cell types. Step 2, the R package “limma” was used to obtain differentially expressed genes (DEGs) in the low- and high-risk score groups and Gene Ontology (GO) enrichment analysis was performed using the clusterProfiler package in R. Step 3 involved the use of the package R “CellChat” to analyze cell-cell interactions. (http://www.cellchat.org/).

### 2.7 Statistical Analysis

Statistical analyses were performed *via* R software (version 3.4.3;http://www.Rproject.org). The clinical characteristics of the development and validation groups were compared *via* the Student’s t-test or the Wilcoxon test. Categorical data were analyzed *via* the *x*^2^ test. We used univariable and multivariable Cox proportional hazards regression analyses with clinical data and a radiomic score to identify parameters that predict 1-year BCR. The nomograms were plotted *via* the RMS package. The discrimination performance of the established models was calculated *via* a receiver operating characteristic (ROC) curve, and the results were compared with those of the Delong nonparametric method. Calibration curves were generated to assess the model calibration. Furthermore, decision curve analysis (DCA) was used to assess the clinical utility of the prediction models by calculating the net benefit when various threshold probabilities were evaluated. The radiomic quality score (RQS) was used to measure the methodological quality of the studies. All analyses were performed *via* two-tailed tests with a significance level of *P* < 0.05.

## 3 RESULTS

### 3.1 Baseline Characteristics of the Participants

Table **1** displays the clinical features of the development group (182 participants; median, interquartile range (IQR) age, 63.1 [55.6--68.1] years) and the validation group (167 participants; median IQR age, 62.1 [54.3--64.5] years). The two cohorts were fairly balanced (*P* values varied from 0.107--0.871). The median IQR follow-up time for all patients was 41.4 (37.2--51.7) months. At the 1-year follow-up, 24 patients (13.2%) in the development cohort achieved BCR, whereas 19 patients (11.4%) in the validation cohort achieved BCR.

### 3.2 Reliability of Manual Annotations and Segmentation Accuracy

The Dice score for Mask 1 and Mask 2 was 0.89 ± 0.09. Fig. (**2a**-**c**) show the segmentation results obtained using our method on DWI, which were highly consistent with the ground truth labeled by experts (Mask 2). The 3D U-net had DSC, VOE, and RVD values of 0.91%, 3.13%, and 3.42%, respectively.

### 3.3 Construction of the Radiomic Model

The feature selection included 1226 radiomic features extracted from DWI (Table **S6**). After LASSO regression, 11 features with nonzero coefficients were obtained (Table **S7**) and used to develop a radiomic model (Table **S8**). The distribution of the radiomic scores for each dataset is shown in Fig. (**3**). The radiomic score was greater for the 1-year BCR group than for the non-BCR group in the development cohort (mean, 1.94 ± 0.33 *vs*. −1.32 ± 0.58; *P* <0.05) and validation cohort (mean, 2.24 ± 0.45 *vs*. −1.81 ± 0.51; *P* <0.05). The optimal cutoff value for the radiomic score was 1.0. Patients were divided into two groups: low-risk score (score < 1.0) and high-risk score (score ≥ 1.0).

### 3.4 Clinical-radiomic Nomogram for 1-year BCR Prediction

**Fig. (4):**
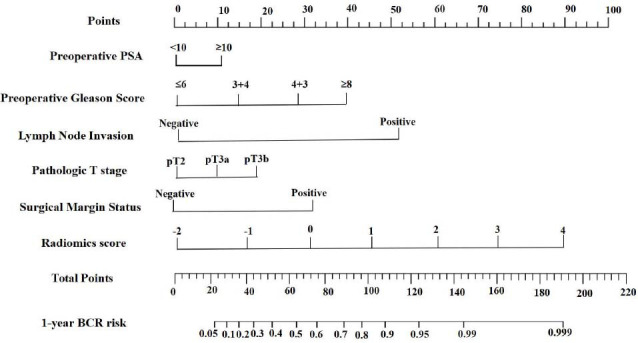
A clinical-radiomic nomogram was developed. A nomogram was established by collecting radiomic, clinical, and pathological data.

Clinical features were significantly different between the 1-year BCR and non-BCR groups according to univariate Cox proportional hazards regression (Table **S9**). Univariate and multivariate Cox analyses were performed to identify independent clinical and radiomic predictors of 1-year BCR in individuals with PR (Table **2**), and a clinical-radiomic nomogram was constructed (Fig. **4**). In both the development and validation groups, the clinical-radiomic nomogram considerably increased the diagnostic accuracy of the clinical and radiomic models (Table **3**). The clinical-radiomic nomogram had AUCs of 0.892 (95% CI: 0.783--0.939) and 0.869 (95% CI: 0.779--0.923) in the development and validation groups, respectively.

### 3.5 Robustness and Clinical Utility Analysis

**Fig. (5):**
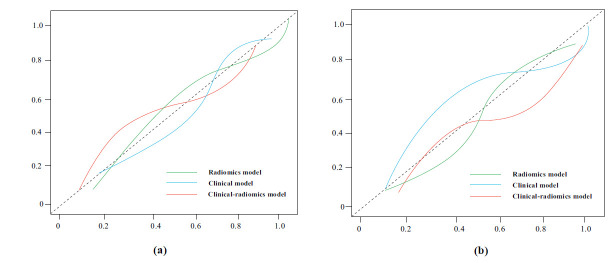
Calibration curves for the established nomograms. (**a**) Calibration curves for the nomograms in the development sets; (**b**) Calibration curves for the nomograms in the validation sets.

**Fig. (6):**
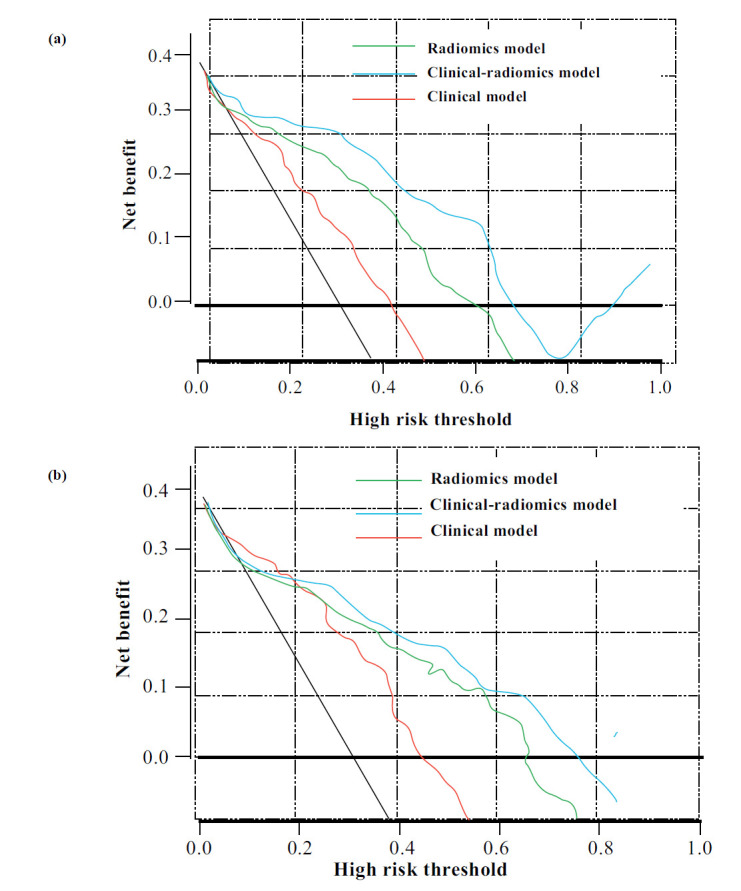
DCA curves of the established nomograms. (**a**) DCA curves of the established clinical-radiomic nomogram compared with the clinical and radiomic models in the development set; (**b**) DCA curves of the established clinical-radiomic nomogram compared with the clinical and radiomic models in the validation set.

(Fig. **5a** and **b**) shows the calibration curves for the nomograms used in both the development and validation groups. The Hosmer-Lemeshow test showed good performance for the clinical-radiomic nomogram in both cohorts (*P* = 0.461 for the development cohort and *P* = 0.722 for the validation cohort). DCA revealed that the use of the clinical-radiomic nomogram provided more value than the use of either the clinical or radiomic score alone when the probability of a clinical decision was greater than 5% for detecting 1-year BCR (Fig. **6a** and **b**). The RQS for this study was 19, suggesting moderate methodological quality (Table **S10**).

### 3.6 Biological Interpretation of the Radiomic Model

**Fig. (7):**
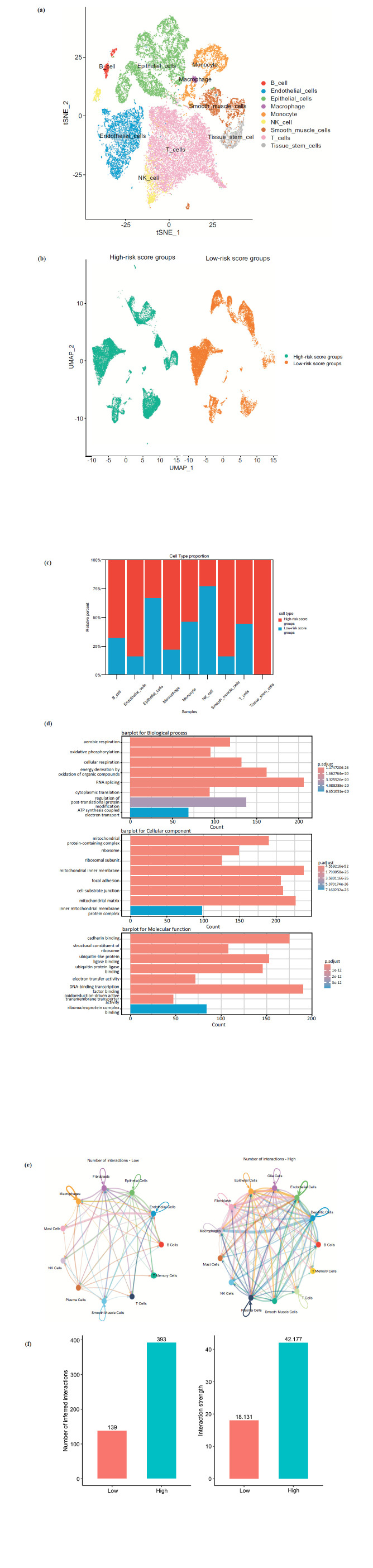
Comparison of the tumor microenvironment between the low- and high-risk score groups. (**a**) A t-distributed stochastic neighbor embedding (tSNE) view of 41,324 single cells, color-coded by assigned cell type. (**b**) Integrative analysis of scRNA-seq samples visualized *via* common UMAP embedding for low- and high-risk score groups. (**c**) Histogram indicating the proportion of cells in the low- and high-risk score groups. (**d**) GO enrichment pathways of the DEGs. (**e**) Network depicting interactions between various cell types in the low- and high-risk score groups. (**f**) Bar plot showing the number and strength of intercellular interactions in both the low- and high-risk score groups.

Single-cell transcriptomic profiles were generated for a total of 41,324 cells, comprising 19,880 cells from the low-risk score group and 21,444 cells from the high-risk score group (Fig. **7a** and **b**). The cellular composition differed significantly between the two groups (Fig. **7c**). Notably, compared with the low-risk score group, the high-risk score group presented 345 upregulated genes and 49 downregulated genes. GO pathway analysis revealed that extracellular matrix (ECM) organization and structural-related pathways were the most significantly enriched pathways in the high-risk score group (Fig. **7d**). Furthermore, receptor-ligand interaction analysis revealed stronger signaling pathway activity and connectivity in the high-risk score group than in the low-risk score group (Fig. **7e** and **f**).

## 4 DISCUSSION

### 4.1 Feasibility of Deep Learning Combined with DWI Radiomics for BCR Prediction

The efficacy of treatment and patient survival after RP depend on the early identification and localization of PCa recurrence [21]. To our knowledge, this is the first study to combine DL analysis *via* DWI-based radiomic data with clinicopathological data to predict the 1-year BCR in patients following RP. We found that the noninvasive DL radiomic technique, along with clinicopathological data, could detect 1-year BCR, with an AUC of 0.892 in the development cohort and 0.869 in the validation cohort. Importantly, our findings obtained *via* scRNA-seq revealed the underlying tumor microenvironment patterns associated with the radiomic score.

### 4.2 Limitations of Existing BCR Prediction Methods

Several methods have been developed to assist in identifying patients who may have BCR [22, 23]. Nonetheless, the efficacy of these methods, which are based on clinicopathological traits, varies among cohorts. Moreover, the prediction power of these models has increased with the advent of mpMRI [24, 25]. Radiomics conveys medical radiologic images into high-throughput quantitative features, thereby offering information about tumor pathogenesis [26]. The potential of radiomic features to predict clinical outcomes has been well established in a range of PCa clinical scenarios. For example, in 2018, Shiradkar *et al*. [27] reported that radiomic features from pretreatment MRI could predict PCa BCR following radical prostatectomy. A recent analysis of 206 PCa patients revealed that the clinical-radiomic model surpassed both the clinical and radiomic models in predicting BCR [28]. However, the generalizability of radiomic signatures remains a significant topic to address, and the varying results could be due to the redundancy caused by handmade radiomic feature extraction. Notably, the traditional interpretation and measurement of PCa relies solely on 2D information from MRI, which ignores the complex 3D structure of the PCa and may result in measurement bias and inconsistency [29, 30]. Total PCa feature evaluation minimizes delineation ambiguity and may thus be sensitive to both micro- and macrorecurrence predictors.

### 4.3 Advantages of Deep Learning-based Segmentation Models

The use of deep learning models for medical image segmentation has significantly increased over the past few decades. Deep learning models can now be used to identify a wide range of anatomical features at the physician level. These models not only minimize workload but also provide an unbiased description of the disease. U-Net and its variants are the most commonly used segmentation layouts and function exceptionally well. Several studies have built specific deep learning-based models based on a convolutional U-Net architecture for autonomous lung, heart, and liver segmentation with encouraging results (DSC values of 0.92--0.95) [31]. However, evaluating interobserver reproducibility is crucial for establishing the stability and robustness of quantitative imaging outcomes for clinical application. To increase the efficiency and inter-operator reliability of data annotation in this study, annotations performed by a junior radiologist were modified by an expert radiologist, and both sets of annotations achieved high Dice scores. These findings support the reliability of manual annotation as a ground truth for prostate lesion segmentation. Most importantly, in our study, 3D U-Net was used for PCa segmentation, and the DSC, VOE, and RVD values for target lesion segmentation were 0.91%, 3.13%, and 3.42%, respectively, demonstrating that the 3D U-Net algorithm can accurately recognize and segment PCa from DWI images.

### 4.4 Role of Clinical and Radiomic Features in BCR Prediction

In the present study, we employed both univariate and multivariate analyses to determine clinical factors. The results demonstrated that a greater preoperative PSA level, preoperative GS, pathological T stage, lymph node invasion, and positive surgical margin status could be used to predict BCR from a clinical standpoint. This conclusion is consistent with that of previous studies [32, 33]. The radiomic signature and aforementioned clinical indicators were integrated to develop a novel nomogram for early BCR prediction. This study revealed that the most promising method to help specialists make more individualized treatment decisions might be the combined clinical-radiomic model, which outperforms the clinical and radiomic features separately and has the highest AUC and greatest net benefit across all threshold probabilities in the DCA.

### 4.5 Biological Significance of Radiomic Scores

Current evaluation of the tumor microenvironment relies heavily on invasive pathological methods, limiting its clinical applicability. To overcome this, radiomic-based approaches have emerged as noninvasive tools for TME characterization. For example, Wang *et al*. [34] employed radiogenomic analysis to validate a radiomic model that deciphers intratumoral heterogeneity in hepatocellular carcinoma (HCC) with microvascular invasion (MVI), focusing on immune cell infiltration and metabolic reprogramming. Feng *et al*. [26] utilized CT radiomics to predict the macrotrabecular-massive subtype and immune status in HCC, emphasizing structural imaging features. Our study builds on these findings by revealing distinct TME characteristics between low- and high-risk score groups. We first analyzed the cell type composition, identifying substantial differences between the groups. Second, we demonstrated that differentially expressed genes (DEGs) were predominantly associated with extracellular matrix (ECM) organization and structural pathways, including cellular functions and metabolic regulation. Notably, gene upregulation/downregulation patterns varied significantly by risk score, underscoring the biological basis of MRI radiomic scores. Finally, CellChat analysis highlighted divergent signaling pathways between the risk score groups, revealing significant variability in signal intensity and cellular communication processes.

### 4.6 Study Quality Assessment and Methodological Considerations

RQS is a recently developed methodology for assessing the rigorous methodology of radiomic research [35]. The RQS for this study was 19, which is acceptable compared with earlier publications. The main strengths of our study are that we segmented the entire 3D tumor volume for radiomic analysis and the multi-institutional design; however, the disadvantage of the present study is that we failed to investigate the repeatability of radiomic features at multiple time points.

### 4.7 Limitations and Future Directions

The current study had several limitations. First, because this was a retrospective study, selection bias was unavoidable. Therefore, multicenter prospective studies are needed. Second, the cohorts had a small sample size, and larger sample sizes for radiotranscriptomic analysis are needed in the future. Third, our identification of radiomic pathway linkages was hypothesis-generating.

## CONCLUSION

In conclusion, this study highlights the feasibility of integrating a DWI-based clinical-radiomic nomogram with deep learning for 1-year BCR prediction, revealing a significant association between radiomic scores and the tumor microenvironment (TME).

## Figures and Tables

**Fig. (2):**
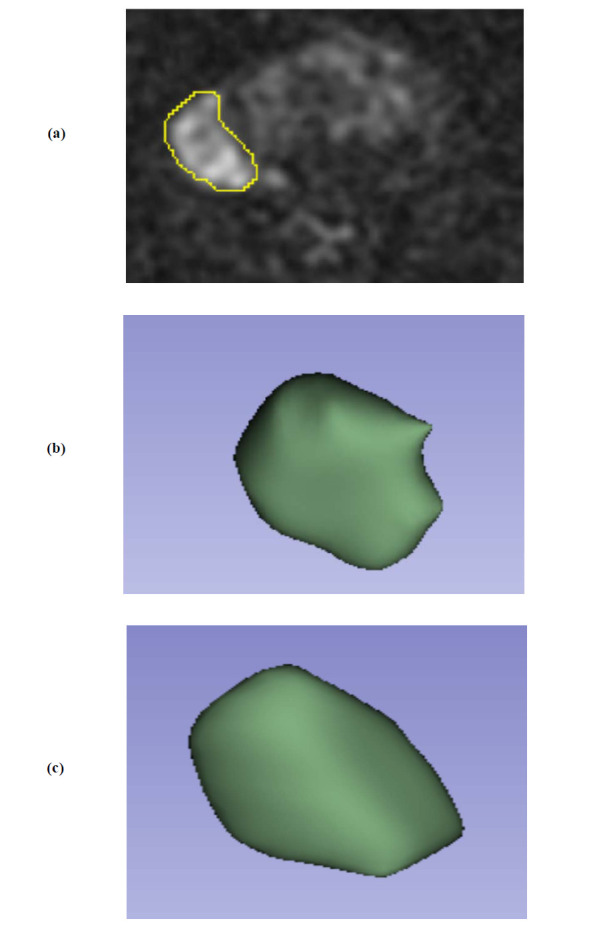
Example of 3D U-Net segmentation for prostate cancer. (**a**) Manual segmentation of a DWI image; (**b**) Ground truth for segmentation results (Mask 2); (**c**) 3D U-Net segmentation results.

**Fig. (3):**
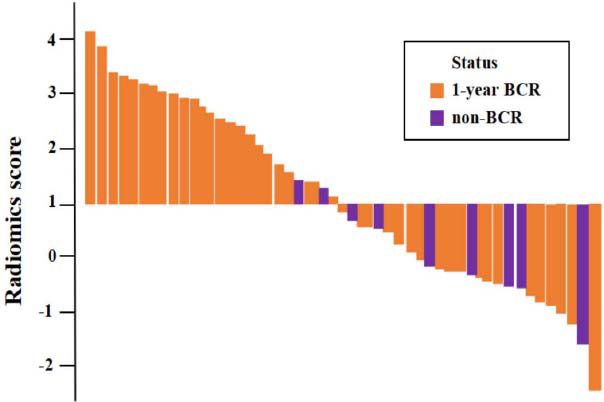
Waterfall plot of the radiomic score distribution for the 1-year BCR and non-BCR groups. The yellow bars represent patients who experienced a 1-year BCR, whereas the purple bars represent patients who did not experience a BCR.

**Table 1:** Patient demographics of the development and validation cohorts.

**Characteristic**	**Development Cohort:** **Institution I (n=182)**	**Validation cohort:** **Institution II (n=167)**	**P-value**
Age, median (IQR), y	63.1 (55.6-68.1)	62.1 (54.3-64.5)	0.613
BMI, median (IQR), kg/m^2^	23.2 (22.1-25.9)	25.1 (21.9-27.3)	0.322
Preoperative PSA, median (IQR), ng/ml	12.4(9.1.6-17.9)	12.3(7.6-17.6)	0.461
Prostate volume, median (IQR), ml	39.7(30.1-49.9)	38.7(31.9-47.2)	0.219
PSA density, median (IQR), ng/ml/cc	0.29 (0.08-1.96)	0.21 (0.11-1.72)	0.107
Preoperative Gleason Score	-	-	0.234
≤6	39	32	-
3+4=7	56	50	-
4+3=7	67	61	-
≥8	20	24	-
lymph node invasion	-	-	0.329
Positive	24	29	-
Negative	158	138	-
Pathologic T stage	-	-	0.674
pT2	101	92	-
pT3a	60	52	-
pT3b	21	23	-
Surgical margin status	-	-	0.332
Positive	61	65	-
Negative	121	102	-
1-year BCR	-	-	0.871
Present	24	19	-
Absent	158	148	-

**Abbreviations:** IQR, interquartile range; BCR, biochemical recurrence; BMI, body mass index; PSA, prostate-specific antigen.

**Table 2:** Univariable and multivariable Cox proportional hazard regression analyses were performed to identify factors associated with 1-year biochemical recurrence.

**Covariate**	**Cox univariable analysis**		**Cox multivariable analysis**	
	**HR (95% CI)**	**P-value**	**HR (95% CI)**	**P-value**
Age	0.723 (0.432–1.432)	0.132		
BMI	1.556 (0.864–2.331)	0.298		
Preoperative PSA	1.874 (1.228–2.874)	<0.001	2.982 (1.890–3.578)	<0.001
Prostate volume	1.561 (1.045–2.981)	0.204		
PSA density	1.568 (0.973–2.832)	0.132		
Preoperative Gleason Score	1.789 (1.241–2.891)	<0.001	1.567 (1.201–3.327)	<0.001
Lymph node invasion	2.456 (1.818–5.221)	<0.001	3.451 (1.781–6.326)	<0.001
Pathologic T stage	2.231 (1.326–5.234)	<0.001	4.323 (2.345–7.423)	<0.001
Surgical margin status	2.714 (1.341–4.532)	0.018	2.778 (1.458–4.671)	<0.001
Radiomic score	3.981 (1.456–5.437)	<0.001	4.108 (2.941–6.649)	<0.001

**Abbreviations:** HR, hazard ratio; CI, confidence interval; BMI, body mass index; PSA, prostate-specific antigen.

**Table 3:** Results of the ability of the radiomic score, clinical nomogram, and clinical-radiomic nomogram to predict 1-year biochemical recurrence.

	**Development Cohort**				**Validation cohort**			
Comparison	AUC (95% CI)	P-value	Sensitivity	Specificity	AUC (95% CI)	P-value	Sensitivity	Specificity
Radiomic score	0.812 (0.774-0.867)	0.014	0.853	0.726	0.792 (0.754-0.851)	0.009	0.834	0.741
Clinical nomogram	0.723 (0.704-0.821)	<0.001	0.785	0.812	0.752 (0.706-0.821)	<0.001	0.814	0.723
Clinical-radiomic nomogram	0.892 (0.783-0.939)		0.892	0.849	0.869 (0.779-0.923)		0.833	0.889

**Abbreviations:** AUC, area under the curve; CI, confidence interval. P-value indicates the significance of the differences in AUCs between the clinical-radiomic model and the other models.

## Data Availability

All the data and supporting information are provided within the article.

## LIST OF ABBREVIATIONS

DL: Deep learning
BCR: Biochemical recurrence
PCa: Prostate cancer
RP: Radical prostatectomy
MRI: Magnetic resonance imaging
DWI: Diffusion-weighted imaging
PI-RADS: Prostate Imaging Reporting and Data System
RQS: Radiomic quality score
CAPRA: Prostate cancer risk assessment score
CNN: Convolutional neural network
GS: Gleason score
PSA: Prostate-specific antigen
LASSO: Least absolute shrinkage and selection operator
DSC: Dice similarity coefficient
VOE: Volumetric overlap error
RVD: Relative volume difference
ROC: Receiver operating characteristic
DCA: Decision curve analysis
IQR: Interquartile range
CI: Confidence interval
TME: Tumor microenvironment

## STANDARDS OF REPORTING

STROBE guidelines were followed.
