# Supplementary material for: DWI-based Biologically Interpretable Radiomic Nomogram for Predicting 1-year Biochemical Recurrence after Radical Prostatectomy: A Deep Learning, Multicenter Study
Source: Curr Med Imaging. 2025 Jun 10;21:e15734056403104. doi: 10.2174/0115734056403104250527045320 (PMC13223430; doi:10.2174/0115734056403104250527045320)
Supplement: Supplementary file 1 [file CMIM-21-E15734056403104_SD1.pdf]

1573-4056/25

Send Orders for Reprints to reprints@benthamscience.net

BENTHAM SCIENCE

CrossMark

Current Medical Imaging

Content list available at: https://benthamscience.com/journals/cmir

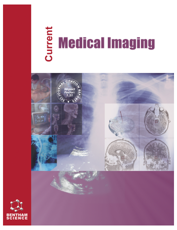

Supplementary Material

DWI-based Biologically Interpretable Radiomic Nomogram for Predicting 1-year Biochemical Recurrence after Radical Prostatectomy: A Deep Learning, Multicenter Study

Xiangke Niu[1,2], Yongjie Li[3], Lei Wang[4] and Guohui Xu[1,*]

[1]*Department of Interventional Radiology, Sichuan Cancer Hospital and Institute, Sichuan Cancer Center, School of Medicine, University of Electronic Science and Technology of China, Chengdu 610041, China*
[2]*Department of Radiology, Affliated Hospital of Chengdu University, Chengdu 610081, Sichuan, China*
[3]*MOE Key Lab for Neuroinformation, University of Electronic Science and Technology of China, Chengdu 610054, China*
[4]*Department of Radiology, Ninety-three Hospital, Jiangyou City 621700, Sichuan, China*

Article History	Received: April 08, 2025	Revised: May 09, 2025	Accepted: May 14, 2025

Table S1. Imaging parameters.

Imaging parameters
All images from the MRI at different cohorts were obtained with 1.5T and 3.0T MRI scanners (Magnetom Skyra, Siemens Healthcare, or Discovery MR 750, GE). Diffusion-weighted imaging was performed utilizing spin–echo sequence, single-shot echoplanar read-out, monopolar diffusion gradient technique, and the following parameters: repetition time/echo time (TR/TE) of 3141 ms/51 ms, a field of view (FOV) of 250×250 mm2, an acquisition matrix of 100×99, a reconstruction matrix of 224×224, a slice thickness of 5 mm, three diffusion directions per b-value, diffusion gradient timing (Δ) of 24.5 ms, diffusion gradient duration (δ) of 12.6 ms, diffusion time (Δ−δ/3) of 20.3 ms, and six b-values of 0, 700, 900, 1100, 1500, and 2000 s/mm2. Patient preparation comprised a 6-hour fast, bowel preparation two hours before testing, and bladder emptying. To reduce peristalsis, 1 mL of scopolamine butylbromide (Buscopan, Boehringer Ingelheim, Ingelheim, Germany) was administered as a slow bolus infusion at 20 mg/mL and diluted in 10 mL of saline.

Table S2. Image preprocessing and segmentation.

Image preprocessing and segmentation
The MRI was preprocessed using a pipeline to normalize intensity and geometric variances between patients. All DICOM pictures were converted to the NIfTI format before being processed, analyzed, and trained, keeping the original spatial resolution. Diffusion-weighted series (ADC and high b-value) were resampled to match the spatial resolution of T2W imaging. The contours were converted to NIfTI format as binary masks with the same spatial resolution as the T2W images. Images were tagged manually with 3D Slicer version 4.11. The area of the target lesion on each DWI image was manually demarcated by two radiologists.

Table S3. 3D U-Net model.

3D U-Net model
The 3D U-Net CNN was developed for prostate cancer segmentation on DWI images by replacing all 2D processes (convolution kernels, pooling layers, and upconvolution kernels) in the U-Net architecture with their 3D counterparts. By fully utilizing 3D spatial information, the algorithm can learn typical features that have higher discriminative power than 2D CNNs. The 3D U-Net was trained on DWI images and their manual annotations. All input images were downsized to 64 × 256 × 256 (z, y, x) before training. This was done to maintain the best image features. To train the 3D U-Net segmentation models, we utilized the ADAM optimizer with an initial learning rate of 10-4 and a fixed batch size of 10 images. For data augmentation, the images were skewed (angel: 0-5), sheared (angel: 0-5), and translated (scale: -0.1, 0.1). U-Net was trained for 300 epochs until the validation loss function failed to rise. The network was developed in Python (version 3.6). Training and validation were carried out using the deep learning framework PyTorch (version 0.4.1). All studies were carried out on an NVIDIA Tesla P100 16GB running Ubuntu.

DOI: 10.2174/0115734056403104250527045320, 2025, *21*, e15734056403104

Table S4. Radiomic feature extraction.

**Radiomic feature extraction**
The input DWI was used to generate b-value images at b = 1000 s/mm2, and radiomic features were extracted from the automatically segmented target volumes using Python 3.7.0 and PyRadiomics 2.2.0. Using the feature extraction tool, we extracted 1226 radiomic features from each target lesion's DWI images. These radiomic features can be divided into seven categories: (1) Shape features (n=8); (2) First-order statistics (n=13); (3) Gray-level co-occurrence matrix (GLCM) features (n=23); (4) Gray-level run length matrix (GLRLM) features (n=16); (5) Gray-level size zone matrix (GLSZM) features (n=16); (6) Gray-level dependence matrix (GLDM) (n=14); and (7) Neighboring Gray Tone Difference Matrix (NGTDM) features (n=5). After 5 wavelet filter transformations and 8 Gaussian Laplace (LoG) transformations, all characteristics except shape features rise by 14 times (1+5+8). Thus, the total number of features collected at each stage is: 8*1 (shape features) + (13+23+16+16+14+5)*14 = 1226.

Table S5. scRNA-seq data collection and analysis.

Single-cell sequencing data collection and processing	For quality control, the raw gene expression matrix was filtered, normalized using the Seurat R package, and selected according to the following criteria: cells with > 1,000 UMI counts, > 200 genes and< 6,000 genes, and< 20% mitochondrial gene expression in UMI counts. Gene expression matrices from filtered cells were normalized and scaled. The uniform manifold approximation and projection (UMAP) method was used to lower the dimensions of the data, and t-distributed stochastic neighbor embedding (t-SNE) projection was applied to cluster and visualize the results. The cells were annotated using canonical cell surface markers.
Identity the differentially expressed genes (DEGs) in low- and high-risk groups	The R package MAST was used to perform all single-cell differential gene expression analyses and Likelihood ratio tests was performed to identify DEGs between two groups. Benjamini–Hochberg multiple testing correction was used to estimate p.adjust. We consider Genes with FDR<5% were significantly differentially expressed. To detect cluster marker genes, cells from each cluster were compared against all other cells in the experiment. The MAST algorithm was used for statistical testing via the Seurat wrapper function FindAllMarkers, with default parameters for filtering out genes below a minimum logfc of 0.25.
Functional enrichment analysis	The R package 'clusterProfiler' was used to explore the biological attributes of the DEGs. Gene Ontology (GO) pathway enrichment analysis was conducted.
Cell–cell communication analysis	CellChat" in R package was used to perform cell–cell communication analysis (http://www.cellchat.org/). CellChat infers and analyzes intercellular communication networks from scRNA-seq data using network analysis and pattern recognition based on manually curated databases that consider known structural compositions of ligand–receptor interactions. Seurat objects, including count matrix and clustering results for each dataset, were imported to CellChat.

Table S6. Radiomic features extracted.

**Feature Classes**		**Feature Names**
Shape Features		Compactness 1, Compactness 2, Spherical Disproportion, Mesh Volume, Sphericity, Surface Area, Surface Volume Ratio, Voxel Volume
Intensity Features		Maximum, Median, Minimum, Mean, Energy, Entropy, Variance, Kurtosis, Root Mean Square, Skewness, Standard deviation, Mean Absolute Deviation, Uniformity
Texture Features	GLCM	Contrast, Correlation, Autocorrelation, Cluster Tendency, Sum Average, Sum Entropy, Sum Squares, Difference Average, Difference Variance, Difference Entropy, Cluster Prominence, Cluster Shade, Maximum Probability, Inverse Difference Moment, Informational Measure of Correlation 1/2, Inverse Difference Moment Normalized, Inverse Difference Normalized, Inverse Difference, Inverse Variance, Maximal Correlation Coefficient, Joint Average, Joint Energy, Joint Entropy
	GLDM	Dependence Entropy, Dependence Non-Uniformity, Dependence Non-Uniformity Normalized, Dependence Variance, Gray-Level Non-Uniformity, Gray-Level Variance, High Gray-Level Emphasis, Large Dependence Emphasis, Large Dependence High Gray-Level Emphasis, Large Dependence Low Gray-Level Emphasis, Low Gray-Level Emphasis, Small Dependence Emphasis, Small Dependence High Gray-Level Emphasis, Small Dependence Low Gray-Level Emphasis
	GLRLM	Gray-Level Nonuniformity, Gray-Level Nonuniformity Normalized, Gray-Level Variance, High Gray-Level Run Emphasis, Long Run Emphasis, Long Run High Gray-Level Emphasis, Long Run Low Gray-Level Emphasis, Low Gray-Level Run Emphasis, Run Entropy, Run Length Non-Uniformity, Run Length Non-Uniformity Normalized, Run Percentage, Run Variance, Short Run Emphasis, Short Run High Gray-Level Emphasis, Short Run Low Gray-Level Emphasis
	GLSZM	Gray-Level Non-Uniformity, Gray-Level Non-Uniformity Normalized, Gray-Level Non-Uniformity Normalized, High Gray-Level Zone Emphasis, Large Area Emphasis, Large Area High Gray-Level Emphasis, Large Area Low Gray-Level Emphasis, Low Gray-Level Zone Emphasis, Size Zone Non Uniformity, Size Zone Non-Uniformity Normalized, Small Area Emphasis, Small Area High Gray-Level Emphasis, Small Area Low Gray-Level Emphasis, Zone Entropy, Zone Percentage, Zone Variance
	NGTDM	Coarseness, Contrast, Busyness, Complexity, Strength

Table S7. Optimal features in the radiomic model.

	Radiomic Features	Coefficient
f1	GLCM.Inverse Difference Moment Normalized	0.2645
f2	GLCM.Maximal Correlation Coefficient	-0.0341
f3	GLDM.Large Dependence Emphasis	-0.067
f4	GLDM.Small Dependence High Gray-Level Emphasis	0.6781
f5	Wavelet_HHH.GLSZM.Short Run Low Gray-Level Emphasis	0.7823
f6	Wavelet_HLH.NGTDM.Contrast	-0.0027
f7	Wavelet_HLH.GLRLM.Gray-Level Non-Uniformity	-0.2671
f8	Wavelet_LHH.GLSZM.Large Area High Gray-Level Emphasis	-0.0031
f9	Wavelet_HLH.GLRLM.Run Length Non-Uniformity	0.0012
f10	LoG sigma-1-0-mm.GLRLM.Short Run High Gray-Level Emphasis	0.0021
f11	LoG sigma-3-0-mm.GLSZM.Large Area High Gray-Level Emphasis	0.0932

Table S8. Radiomic model construction.

**Radiomic model construction**
The radiomic signature, or the RadRisk score was calculated as a linear combination of the 11 selected features weighted by their nonzero LASSO coefficients as the Radiomic score $=-2.6767+f1\times0.2645-f2\times0.0341-f3\times0.067+f4\times0.6781+f5\times0.7823-f6\times0.0027-f7\times0.2671-f8\times0.0031+f9\times0.0012+f10\times0.0021+f11\times0.0932$

Table S9. Univariate and multivariate Cox proportional hazards regression analyses showing predictors of 1-year biochemical recurrence.

**Covariate**	**Cox univariable analysis**		**Cox multivariable analysis**	
	**HR (95% CI)**	***P* value**	**HR (95% CI)**	***P* value**
Age	0.524 (0.228–1.436)	0.132	-	-
BMI	1.568 (0.789–2.437)	0.224	-	-
Preoperative PSA	1.962 (1.1188–2.987)	<0.001	2.451 (1.780–3.452)	<0.001
Prostate volume	1.234 (0.674–1.879)	0.359	-	-
PSA density	1.418 (0.876–2.427)	0.156	-	-
Preoperative Gleason Score	1.923 (1.457–3.342)	<0.001	2.378 (1.673–3.675)	<0.001
lymph node invasion	2.890 (1.984–5.986)	<0.001	2.468 (1.893–6.560)	<0.001
Pathologic T stage	2.561 (1.785–5.568)	<0.001	3.563 (2.345–5.457)	<0.001
Surgical margin status	2.456 (1.341–4.098)	0.025	2.541 (1.235–3.451)	<0.001

Abbreviations: HR, hazard ratio; CI, confidence interval; BMI, body mass index; PSA, prostate-specific antigen.

Table S10. Methodological quality was evaluated via the radiomic quality score (RQS).

**Criteria**		**Points**
1	Image protocol quality-well-documented image protocols (for example, contrast, slice thickness, energy, etc.) and/or usage of public image protocols allow reproducibility/replicability	2
2	Multiple segmentations-possible actions are: segmentation by different physicians/algorithms/software, perturbing segmentations by (random) noise, segmentation at different breathing cycles. Analyze feature robustness to segmentation variabilities	1
3	Phantom study on all scanners-detect interscanner differences and vendor-dependent features. Analyze feature robustness to these sources of variability	0
4	Imaging at multiple time points-collect images of individuals at additional time points. Analyze feature robustness to temporal variabilities (for example, organ movement, organ expansion/shrinkage)	0
5	Feature reduction or adjustment for multiple testing-decreases the risk of overfitting. Overfitting is inevitable if the number of features exceeds the number of samples. Consider feature robustness when selecting features	3
6	Multivariable analysis with non radiomics features (for example, EGFR mutation)-is expected to provide a more holistic model. Permits correlating/inferencing between radiomics and non radiomics features	1
7	Detect and discuss biologic correlates-demonstration of phenotypic differences (possibly associated with underlying gene–protein expression patterns) deepens understanding of radiomics and biology	1

8	Cutoff analyses-determine risk groups by either the median, a previously published cutoff or report a continuous risk variable. Reduces the risk of reporting overly optimistic results	1
9	Discrimination statistics-report discrimination statistics (for example, C-statistic, ROC curve, AUC) and their statistical significance (for example, P values, confidence intervals). One can also apply resampling method (for example, bootstrapping, cross-validation)	2
10	Calibration statistics-report calibration statistics (for example, Calibration-in-the-large/slope, calibration plots) and their statistical significance (for example, P values, confidence intervals). One can also apply resampling method (for example, bootstrapping, cross-validation)	1
11	Prospective study registered in a trial database-provides the highest level of evidence supporting the clinical validity and usefulness of the radiomics marker	0
12	Validation-the validation is performed without retraining and without adaptation of the cutoff value, provides crucial information with regard to credible clinical performance	2
13	Comparison to 'gold standard'-assess the extent to which the model agrees with/is superior to the current 'gold standard' method (for example, TNM-staging for survival prediction). This comparison shows the added value of radiomics	1
14	Potential clinical utility-report on the current and potential application of the model in a clinical setting (for example, decision curve analysis).	2
15	Cost-effectiveness analysis-report on the cost-effectiveness of the clinical application (for example, QALYs generated)	0
16	Open science and data-make code and data publicly available. Open science facilitates knowledge transfer and reproducibility of the study	2
**RQS score (%) =**		52.7
**Total points (36 = 100%)**		19

© 2025 The Author(s). Published by Bentham Science Publisher.

This is an open access article distributed under the terms of the Creative Commons Attribution 4.0 International Public License (CC-BY 4.0), a copy of which is available at: https://creativecommons.org/licenses/by/4.0/legalcode. This license permits unrestricted use, distribution, and reproduction in any medium, provided the original author and source are credited.
